# Rapid direct conversion of bovine non-adipogenic fibroblasts into adipocyte-like cells by a small-molecule cocktail

**DOI:** 10.3389/fcell.2023.1020965

**Published:** 2023-02-02

**Authors:** Longfei Sun, Dandan Zhang, Liangshan Qin, Quanhui Liu, Guodong Wang, Deshun Shi, Ben Huang

**Affiliations:** ^1^ State Key Laboratory for Conservation and Utilization of Subtropical Agro-Bioresources, School of Animal Science and Technology, Guangxi University, Nanning, Guangxi, China; ^2^ Guangxi Academy of Medical Science, Nanning, Guangxi, China

**Keywords:** small-molecule compounds, rapid, direct conversion, bovine, fibroblasts to adipocytes

## Abstract

**Introduction:** The molecular regulation mechanism of fat deposition in bovine and its improvement on beef quality are important research directions in the livestock industry. The research of molecular mechanisms that govern the regulation and differentiation of adipocytes may conduct to understand the mechanism of obesity, lipid disorders, and fat deposition. In the recent decade, small-molecule compounds have been widely used in reprogramming and transdifferentiation fields, which can promote the induction efficiency, replace exogenous genes, or even induce cell fate conversion alone. Furthermore, small-molecule compound induction is expected to be a novel approach to generate new cell types from somatic cells *in vitro* and *in vivo*.

**Methods:** In this study, we established rapid chemically induced platform for transdifferentiation of bovine ear fibroblasts into adipocyte-like cells using a small-molecule cocktail (Repsox, VPA, TTNPB). The chemically induced adipocytes (CiADCs) were characterized by lipid staining, qRT-PCR and WB. Bovine natural adipocytes were used as positive control, and the expression of adipocyte-related marker genes in CiADCs were analyzed. Moreover, RNA-Seq explore the mechanism of RVB in the regulation of Bovine adipocyte transdifferentiation.

**Results:** In this study, the chemically induced adipocytes (CiADCs) could be identified as early as day 6. The CiADCs appeared to be circular and rich of lipid droplets. The adipocyte-specific genes of LPL, PPARγ, IGF1, GPD1, C/EBPδ, ADIPOQ, PCK2, FAS, C/EBPβ, PPARGC1A, C/EBPα, and CFD were detected to be significantly upregulated in both CiADCs and natural adipocytes. Western blot analysis also confirmed the increase C/EBPα and PPARγ protein level in induced adipocytes (CiADCs-6d) treated with RVB. In addition, we also found that the signaling pathways (PPAR signaling pathway, PI3K-Akt signaling pathway, p53 signaling pathway, MAPK signaling pathway, and ECM-receptor interaction) regulated by the DEGs played a vital role in adipogenesis.

**Discussion:** In the present study, a combination of small-molecule compounds RVB was used to transdifferentiate bovine ear fibroblasts into the chemically-induced adipocyte cells (CiADCs) that have a large number of lipid droplets. Importantly, the small-molecule cocktail significantly shortened the reprogramming turnaround time. The morphology of CiADCs is close to the “ring type” of natural differentiated adipocytes on sixth day. And, the CiADCs showed similar adipocyte-specific gene expression patterns to natural adipocytes. Furthermore, RVB increased protein expression of PPARγ and C/EBPα in the chemically-induced adipocytes (CiADCs-6d). Our findings reveal that the signaling pathways of C/EBPα and PPARγ play pivotal roles in this transdifferentiation process. In addition, we also found that the signaling pathways (PPAR signaling pathway, PI3K-Akt signaling pathway, p53 signaling pathway, MAPK signaling pathway, and ECM-receptor interaction) regulated by the DEGs played a vital role in adipogenesis. In general, this study provides valuable evidence to deepen our understanding of the molecular mechanism of small molecule cocktails in regulating adipogenesis.

## 1 Introduction

The palatability profile of marbled beef is mainly determined by the content of intramuscular fat (IMF) (Joo et al., 2017; Choi et al., 2019). Relative to the long breeding cycle and the problem of diet-mediated fat deposition effects, direct regulating the differentiation of intramuscular fat cells may be more effective in improving the amount of intramuscular fat (Li et al., 2011; Li et al., 2018). As hotpot, the research of molecular mechanisms that govern the regulation and differentiation of adipocytes may conduct to understand the mechanism of obesity, lipid disorders, and fat deposition (Guo et al., 2020; Sun et al., 2020; Zhou et al., 2021). Transdifferentiation is the conversion of one type of differentiated cell to another type of normally differentiated cells (Cieślar-Pobuda et al., 2017). At present, many types of functional cells have been obtained by transdifferentiation techniques, which have practical value in the design of novel therapies for the treatment of diseases and studying the mechanism of cell differentiation (Yamanouchi et al., 2007; Jia et al., 2019; Zhang et al., 2021).

In the recent decade, small-molecule compounds have been widely used in reprogramming and transdifferentiation fields, which can promote the induction efficiency, replace exogenous genes, or even induce cell fate conversion alone (Cao et al., 2017; Bansal et al., 2019; Zhou and Sun, 2019). Furthermore, small-molecule compound induction is expected to be a novel approach to generate new cell types from somatic cells *in vitro* and *in vivo* (Kim et al., 2020). Previous studies have shown the direct conversion of fibroblasts [human (Takeda et al., 2017; Sowa et al., 2021), mouse (Tu et al., 2019), and pig (Zhu et al., 2012)] into adipocytes using small-molecular compounds. Until now, it has not been well characterized yet about the mechanism of adipose differentiation by transdifferentiation of bovine fibroblasts into adipocyte using small chemical molecules.

In this study, we established a highly efficient and rapid chemically induced platform for transdifferentiation of bovine fibroblasts into adipocyte-like cells using a small-molecule cocktail (Repsox, VPA, TTNPB). The adipocyte-like cells appeared to be circular and rich of lipid droplets and expressed adipocyte-specific genes. RepSox, a small-molecule inhibitor of transforming growth factor-beta receptor I (TGF-β-RI), has been reported to enhance both mouse and human reprogramming (Zhou and Sun, 2019). Repsox can induce adipogenesis in primary mouse fibroblasts and sheep fibroblasts (Tu et al., 2019; Guo Y et al., 2021). The histone deacetylase valproic acid (VPA) is a short-chain carboxylic acid. In MEFs, it increases the expression of pluripotency-related genes which improves four TFs as well as OSK reprogramming efficiency (Zhou and Sun, 2019). TTNPB acts as a potent retinoic acid analogue with RARα/β/γ affinity. It is a component of a set of small molecules for factor-free reprogramming. TTNPB has been shown to enhance the chemical reprogramming efficiency up to a factor of 40 (Baranek et al., 2017). As a differentiated cell model of adipocyte *in vitro*, this study laid the theoretical foundation for subsequent studies on the mechanisms of adipocyte deposition and adipocyte developmental differentiation. Moreover, the method for conversion of fibroblasts into adipocytes induced by small-molecule compounds may provide a novel strategy to increase intramuscular fat for improving beef meat quality.

## 2 Materials and methods

### 2.1 Ethics statement

This study was approved and monitored by the animal experiments ethical review committee of Guangxi University, Nanning, China.

Animal experiments were carried out in accordance with the guidelines on animal care and use established by the Guangxi University Animal Care and Use Committee.

### 2.2 Cell culture

#### 2.2.1 Bovine ear fibroblasts and induced transdifferentiation

The ear margin tissue of bovine was collected from the Siye Farm of Guangxi Animal Husbandry. The ear tissue was minced into pieces (1 mm3) and then stuck evenly to the bottom of the 100 mm dish. When the tissue block is firm, 10 mL DMEM containing 10% fetal bovine serum (FBS) was added into dishes. While the bovine fibroblasts (BEFs) grow out from around the tissue block, we removed the tissue block and then the BEFs were seeded in a ratio of 1:3. The BEFs were passaged at least twice for removing tissue and magazines cell at 37°C, 5% CO_2_ incubator. The culture medium for the BEFs was composed of DMEM supplemented with 10% FBS, penicillin, and streptomycin.

BEFs were seeded into 60-mm dish at 80% confluence. After 24 h, cells were treated for 6 days with induction medium (N2B27 + VBR: 500ug/mL VPA, 1 μM TTNPB, 10 μM RepSox) and subsequently transferred to adipocyte maintenance medium (DMEM/F12 contain insulin, rosiglitazone) for another 6 days. The medium was changed every 2 days.

#### 2.2.2 Isolation of natural adipocytes

Pre-adipocytes were cultured from bovine abdomen as described in a previous study (Grant et al., 2008). The pre-adipocyte culture medium was DMEM/F12 supplemented with 20% FBS, penicillin, and streptomycin. The pre-adipocytes were re-suspended in the pre-adipocyte culture medium and seeded on 12-well plates. After 24 h, the pre-adipocytes were induced differentiation with a differentiation medium (DMEM/F12 containing insulin, DEX, IBMX, and Rosiglitazone) for 2 days. Then, the adipocyte maintenance medium (DMEM/F12 containing insulin, and Rosiglitazone) was changed to continue the culture, and the medium was changed every 2 days until the tenth day.

### 2.3 Oil red O staining

The cells were fixed with 4% paraformaldehyde for 15 min and then stained for 15 min with Oil Red O staining solution (Sigma, O0625). Then, the cells were rinsed with 60% isopropyl alcohol solution and washed three times with sterile water.

### 2.4 qRT-PCR

Total RNA was extracted using Trizol reagent (Life Technologies, 149112) and was reverse transcripted into cDNA using SuperMix (Vazyme, R323-01). The cDNA was mixed with Real-Time PCR Master Mix (Vazyme, Q711-02) and matching probes and primers specific for Bovine β-actin, LPL, PPARγ, IGF1, GPD1, C/EBPδ, ADIPOQ, PCK2, FAS, C/EBPβ, PPARGC1A, C/EBPα and CFD (Table 1). The level (average S.D.) was normalized with respect to the β-actin mRNA level in each sample, and is expressed as a value relative to the control group (BEFs). Data analyses were performed using the 2^−ΔΔCT^ method.

**TABLE 1:** Primers.

Gene		Sequence (5′to 3′)	Product length (bp)
β-Actin (NM_173979.3)	Forward	ACCGCAAATGCTTCTAGG	199
	Reverse	ATCCAACCGACTGCTGTC	
LPL (NM_001075120.1)	Forward	CAG​CCC​CGG​CTT​TGA​TAT​TG	177
	Reverse	AGC​TTT​GCC​AAG​TTT​CAG​CC	
C/EBPβ (NM_176788.1)	Forward	TTC​CTC​TCC​GAC​CTC​TTC​TC	79
	Reverse	CCA​GAC​TCA​CGT​AGC​CGT​ACT	
C/EBPδ (NM_174267.2)	Forward	GTC​CGC​CAT​GTA​CGA​CGA​C	134
	Reverse	CGCCCGCTTTGTGGTTGC	
PPARγ (NM_181024.2)	Forward	AAG​AGC​TGA​CCC​GAT​GGT​TG	152
	Reverse	TGA​GGG​AGT​TGG​AAG​GCT​CT	
C/EBPα (NM_176784.2)	Forward	ATCTGCGAACACGAGACG	73
	Reverse	CCA​GGA​ACT​CGT​CGT​TGA​A	
FAS (NM_001012669.1)	Forward	TAA​GGT​TCA​AAT​TGC​TGC​GT	138
	Reverse	TCC​AGA​GCG​AAG​GAG​AGA​TT	
ADIPOQ (NM_174742.2)	Forward	CCG​TTC​TCT​TCA​CCT​ACG​AC	150
	Reverse	CAT​TGA​CAT​TAT​CTG​CAT​AGA​CCC	
CFD (NM_001034255.2)	Forward	TAC​TCC​TGC​CGG​TGC​TCG​AC	184
	Reverse	CCGCAGATCCGTGAACCC	
IGF1 (XM_005206497.4)	Forward	CAT​CTC​CCA​TCT​CCC​TGG​ATT	163
	Reverse	ATG​TGA​TGG​GCA​TCT​TCA​CCT​TC	
GPD1 (NM_001035354.1)	Forward	CAA​CGA​GGT​GGC​TGA​TGA​GA	173
	Reverse	CCC​CAA​CGG​CCA​CTA​TAT​TCT​TT	
PCK2 (NM_001205594.1)	Forward	CTA​AGT​ACA​ACA​ATT​GCT​GGC​TGG	101
	Reverse	CAC​CGT​GTC​CCG​TTG​AGA​A	
PPARGC1A (XM_024993060.1)	Forward	GCA​GAA​GGC​AAT​TGA​AGA​GCG	186
	Reverse	GCA​GCA​AAA​GCA​TCA​CAG​GT	

### 2.5 RNA-seq

Total RNA was extracted using Trizol reagent kit (Invitrogen, Carlsbad, CA, United States) according to the manufacturer’s protocol. RNA quality was assessed using an Agilent 2100 Bioanalyzer (Agilent Technologies, Palo Alto, CA, United States) and checked using RNase free agarose gel electrophoresis. After total RNA was extracted, eukaryotic mRNA was enriched by Oligo (dT) beads, while prokaryotic mRNA was enriched by removing rRNA by Ribo-Zero TM Magnetic Kit (Epicentre, Madison, WI, United States). Then, the enriched mRNA was fragmented into short fragments using fragmentation buffer and reverse transcripted into cDNA with random primers. Second-strand cDNA were synthesized by DNA polymerase I, RNase H, dNTP, and buffer. Then, the cDNA fragments were purified using QiaQuick PCR extraction kit (Qiagen, Venlo, the Netherlands), end repaired, a base added, and ligated to Illumina sequencing adapters. The ligation products were size-selected by agarose gel electrophoresis, PCR amplified, and sequenced using Illumina Novaseq6000 by Gene *Denovo* Biotechnology Co(Guangzhou, China). The paired-end reads were assigned quality scores and aligned to the reference genome using TopHat v2.1.1. Then, counter files were generated with HTSeq v0.6.1, with a |log2Fold- Change| 1 and an adjusted *p*-value < 0.05 considered to indicate statistical significance. Biological process analysis (GO analysis) (1) and KEGG (2) pathway analysis were performed using differentially expressed genes, and the results were visualized with Blast2GO (3) (http://www.BLAST2go.org/), a Cytoscape plug-in.

### 2.6 Western blot analysis

The cells were lysed in denaturing lysis buffer containing protease inhibitors (RIPA, Bioteke, Beijing, China) for 30 min on ice and centrifuged (12,000 × g) for 10 min at 4°C. Protein concentrations in the lysates were determined by using a BCA protein assay kit (Solarbio, Beijing, China). Exactly, 30 μg of protein were separated on a 12% SDS-PAGE and transferred to a nitrocellulose filter membrane that was blocked with 5% non-fat dried milk in tris-buffered saline containing 0.05% Tween 20 (pH 7.6) for 1 h at 25°C. Subsequently, the membranes were incubated overnight at 4°C with GAPDG (abcam), PPARγ (Bioss), C/EBPα(Abmart). Then, the blot was incubated with a horseradish peroxidase-conjugated secondary anti-body for 1 h at 25°C. The signals were visualized by enhanced chemiluminescence (Bio-Rad, California, United States).

### 2.7 Data analysis

The data were presented as the mean SEM. The statistical analysis was performed using SPSS 15.0 software. *p*< 0.05 was considered significant.

## 3 Results

### 3.1 Small-molecule compounds induce the conversion of BEFs into adipocyte-like cells

In the process of screening small-molecule compounds to induce dedifferentiation of BEFs, we surprisingly obtained adipocyte-like cells with circular in shape by using small-molecule compound combination, including (10 μM RepSox, 500 ug/mL VPA, and 1 μM TTNPB), which had similar phenotype with bovine natural adipocytes.

**FIGURE 1:**
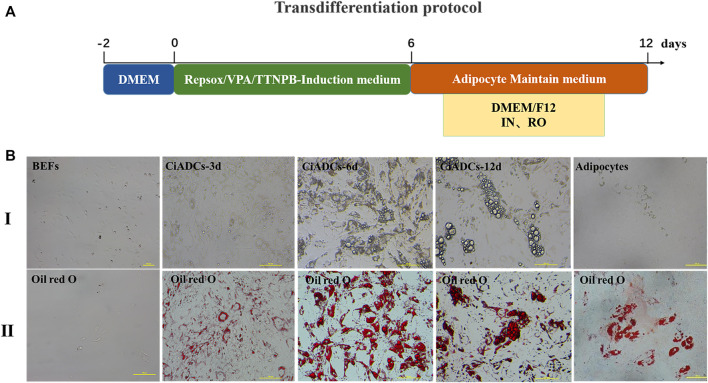
Small-molecule compounds induce the conversion of BEFs into chemically induced adipocyte cells (CiADCs). **(A)** Diagrammatic sketch of CiADC formation. Induction medium was applied from day 0 to day 6, subsequently adipocyte maintain medium was applied. Induced cells were obtained at day 6 termed adipocyte-like cells. **(B)** Phase contrast image and oil red O staining of adipocyte-like cells generated from BEFs during the induction process. I. Morphological changes of CiADCs cells from day 0 to day 12 and differentiated adipocytes; II. The oil red O staining for different induction time points.

The obvious small lipid droplets appeared within induced BEFs for 3 days of post VBR induction. Then, the cell morphology gradually became round and contained a large number of lipid droplets in the cytoplasm at 6 days of post VBR induction. Subsequently, the CiADCs could continue to be cultured with adipocyte maintenance medium. The lipid droplets were positive in oil red O staining (Figure 1).

### 3.2 CiADCs exhibit similar biological properties to natural adipocytes

**FIGURE 2:**
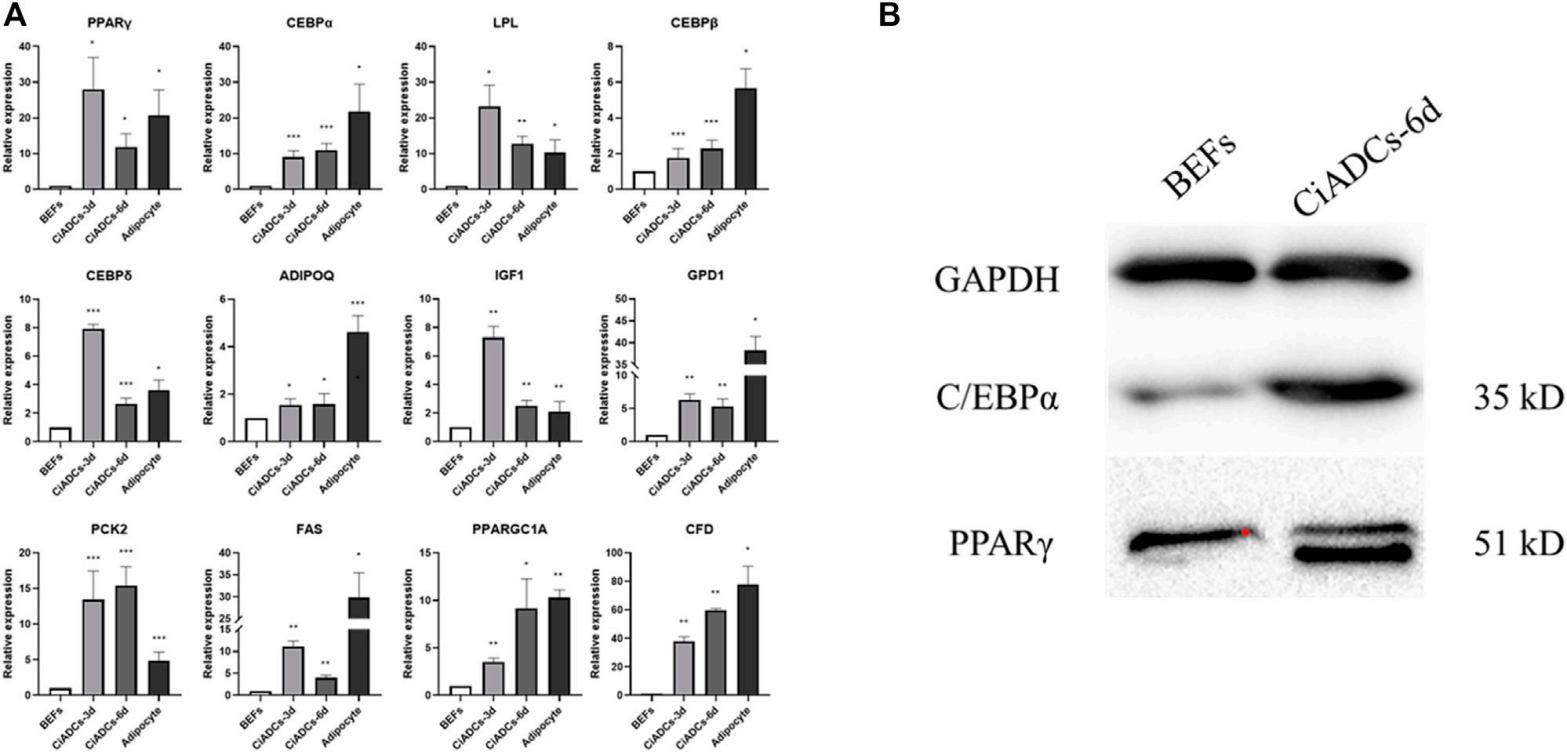
Characterization of adipogenesis induced by RVB in BEFs. **(A)** qRT-PCR analysis the expression of adipogenic marker genes of PPARγ, C/EBPα, LPL, C/EBPβ, C/EBPδ, ADIPOQ, IGF1, GPD1, PCK2, FAS, PPARGC1A, and CFD among CiADCs-3d, CiADCs-6d and natural adipocytes. Values (mean ± SD) are normalized with respect to the β-actin mRNA level in each sample. Significant differences *p < 0.05; **p < 0.01; ***p < 0.001. **(B)**. Western blot analysis of PPARγ and C/EBPα protein levels in induced adipocyte-like cells (CiADCs-6d).

In order to support the abovementioned observations, the expression of adipocyte-specific genes was quantified in the CiADCs-3d, CiADCs-6d, and natural adipocytes. Bovine adipocytes were used as positive control, and the expression of adipocyte-related marker genes in CiADCs was analyzed by qRT-RCR. The results showed that the adipocyte-specific genes of LPL, PPARγ, IGF1, GPD1, C/EBPδ, ADIPOQ, PCK2, FAS, C/EBPβ, PPARGC1A, C/EBPα, and CFD were detected to be significantly upregulated in both CiADCs and natural adipocytes (Figure 2A). Western blot analysis also confirmed the increase C/EBPα and PPARγ protein level in induced adipocytes (CiADCs-6d) treated with RVB (Figure 2B).

### 3.3 Identification of differentially expressed genes enriched in the transdifferentiation into adipocytes

**FIGURE 3:**
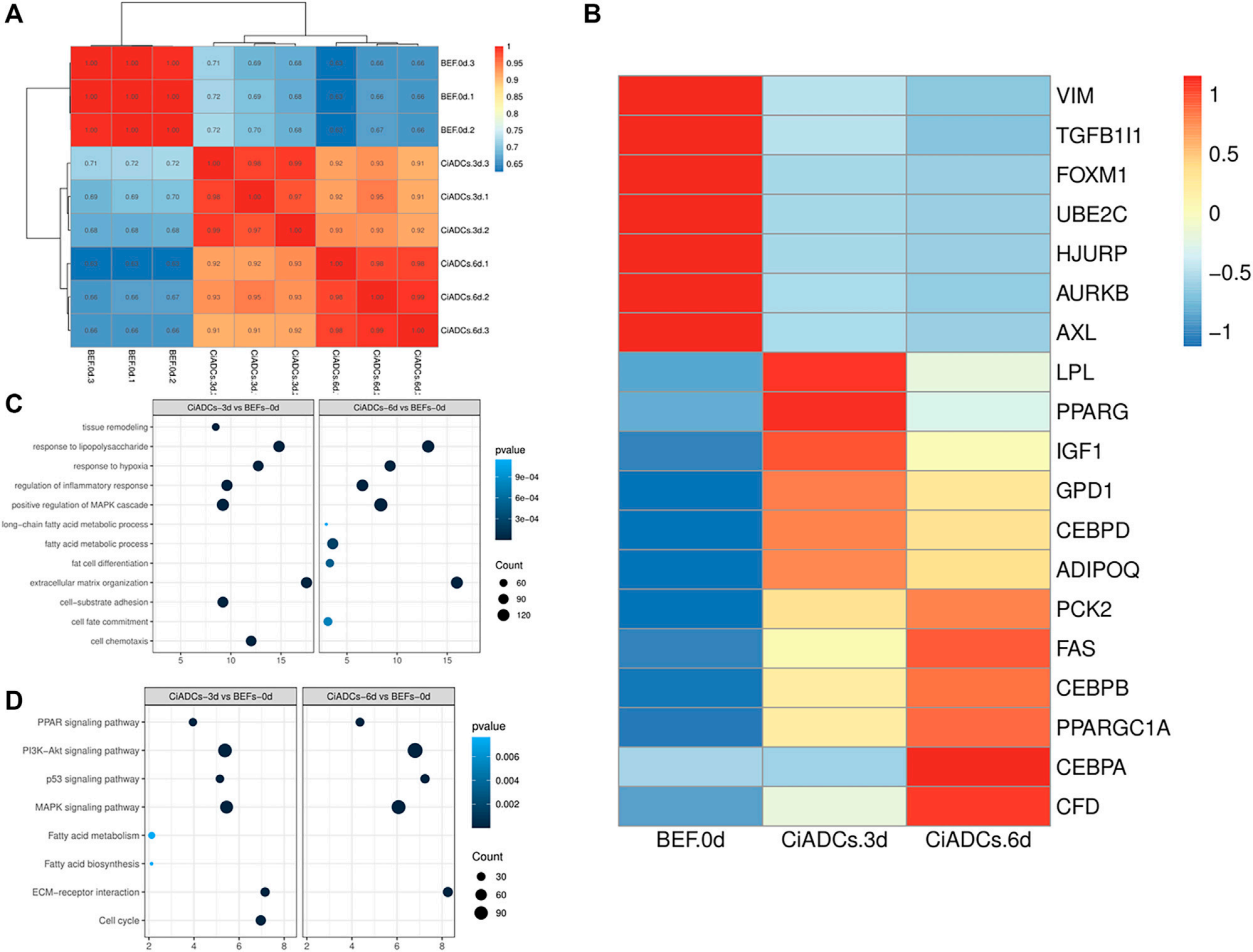
Identification of differentially expressed genes enriched in the transdifferentiation into adipocytes **(A)** Heatmap of BEFs, CiADCs-3d, and CiADCs-6d cells gene expression correlation coefficients in mRNA-seq data. **(B)** Heatmap of the differentially expressed genes. **(C)** GO functional enrichment analysis of the upregulated DEGs. **(D)** KEGG adipogenesis pathway analysis of CiADCs-3d and CiADCs-6d.

To explore the differences in the transdifferentiation process of BEFs induced by small-molecule compounds, we examined chemically induced adipocytes on day 3(CiADCs-3d) and day 6(CiADCs-6d). Correlation analysis proves that the biological repetition is better, and the average value can be used for subsequent analysis. Sequencing results showed that CiADCs-3d/CiADCs-6d has a difference in gene expression pattern from the initial BEFs (Figure 3A). This also reminded that the cell fate of BEFs has changed.

Figure 3B is a heat map of differentially expressed genes, showing the fibroblast genes highly expressed in BEFs and the adipocyte marker genes highly expressed in CiADCs-3d/CiADCs-6d. Moreover, the marker genes of LPL, PPARγ(PPARG), IGF1, GPD1, C/EBPβ(CEBPB), and ADIOPQ have more higher expression in CiADCs-3d. The mRNA levels of marker genes of PCK2, FAS, C/EBPβ(CEBPB), PPARGC1A, C/EBPα(CEBPA), CFD were significantly higher than CiADCs-6d.

GO and KEGG enrichment analysis were conducted to uncover the gene function and biological pathways of DEGs. The GO enrichment analysis (Figure 3C) showed that response to lipopolysaccharide, response to hypoxia, regulation of inflammatory response, positive regulation of MAPK cascade, extracellular matrix organization were significantly enriched in CiADCs-3d/CiADCs-6d. The GO analysis results revealed that the upregulated DEGs were significantly enriched in tissue remodeling, cell-substrate adhesion, and cell chemotaxis in CiADCs-3d. GO of CiADCs-6d analysis revealed that fat adipose specific genes of upregulated were significantly enriched in long-chain fatty acid metabolic process, fatty acid metabolic process, fat cell differentiation, and cell fate commitment. KEGG pathway analysis of CiADCs-3d/CiADCs-6d both annotated significant features related to PPAR signaling pathway, PI3K−Akt signaling pathway, p53 signaling pathway, MAPK signaling pathway, and ECM-receptor interaction, indicating that these substances could also be involved in the mechanism of adipogenesis. When the CiADCs-3d was compared to CiADCs-6d, the significant pathways were fatty acid biosynthesis, fatty acid metabolism, and cell cycle (Figure 3D).

**FIGURE 4:**
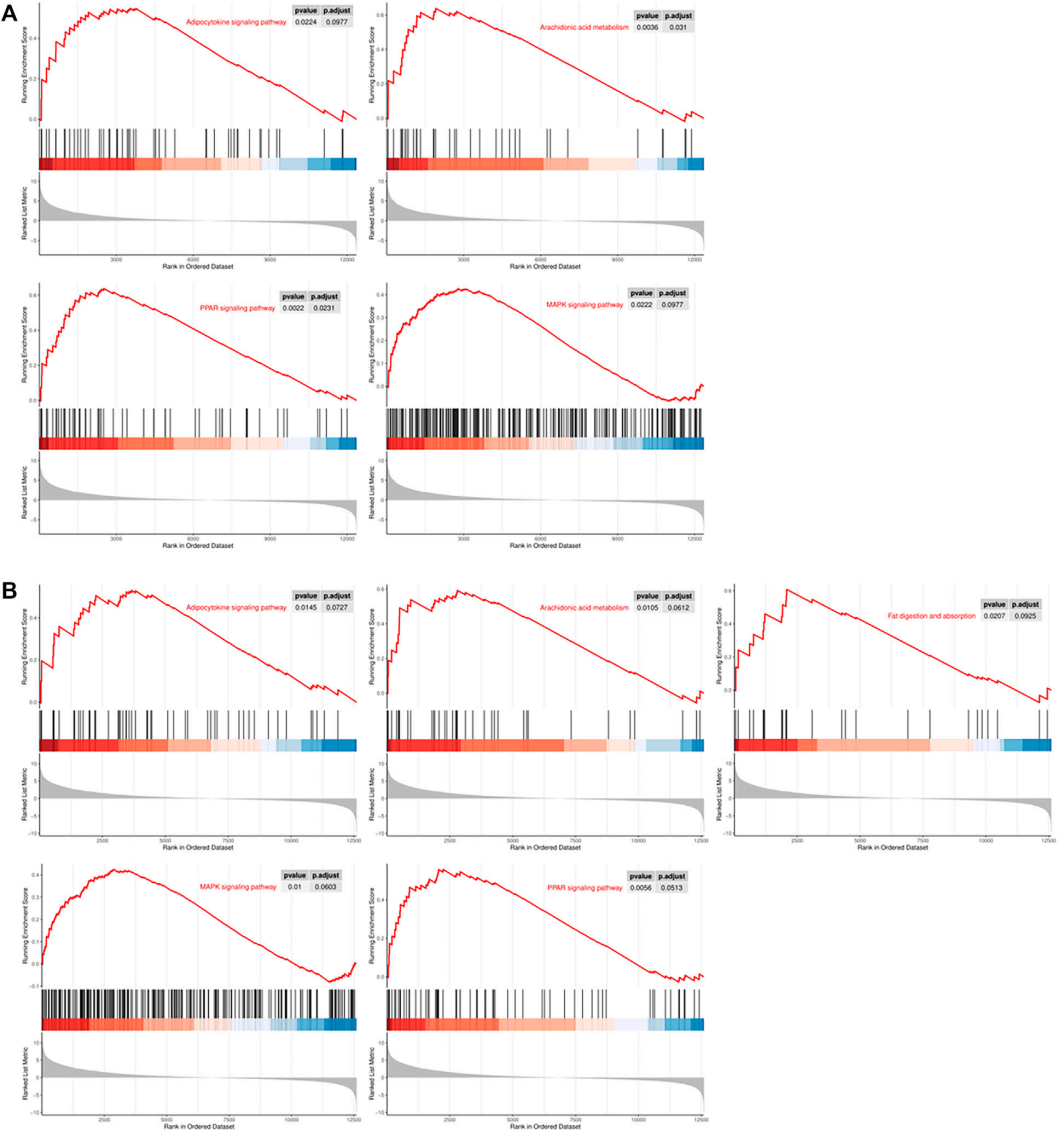
Gene set enrichment analysis (GSEA) of CiADCs-3d/6d revealed several signaling pathways that were either activated **(A)** CiADCs-3d: adipocytokine, arachidonic acid metabolism, MAPK and PPAR signaling pathway. **(B)** CiADCs-6d: adipocytokine, arachidonic acid metabolism, fat digestion and absorption, MAPK and PPAR signaling pathway. Color scaled within each row. Red color indicates high expression level, while blue color indicates low expression level.

Gene set enrichment analysis (GSEA) revealed that numerous adipogenesis-related KEGG pathways were significantly upregulated, which included adipocytokine, arachidonic acid metabolism, MAPK and PPAR signaling pathway (Figure 4). Furthermore, fat digestion and absorption pathways were only upregulated in CiADCs-6d (Figure 4B). Altogether, these findings indicated that the signal pathways of regulating adipose differentiation have been turned on in CiADCs-3d (Figure 4A).

## 4 Discussion

In the present study, a combination of small-molecule compounds RVB was used to transdifferentiate bovine ear fibroblasts into the chemically-induced adipocyte cells (CiADCs) that have a large number of lipid droplets. Importantly, the small-molecule cocktail significantly shortened the turnaround time and enhanced reprogramming efficiency. The morphology of CiADCs is close to the “ring type” of natural differentiated adipocytes on sixth day. qRT-PCR analyses revealed that the adipocyte-specific genes were significantly upregulated. Through the analysis from CiADCs-3d to CiADCs-6d by transcriptome, we found some signaling pathways that regulate the adipose differentiation were switched on continuous. Collectively, our results showed a chemical induction method that efficient induction of conversion into CiADCs, which could contribute to study the mechanisms of adipocyte transdifferentiation and adipocyte developmental differentiation.

Adipocyte-like cell could be identified as early as 6 days and their numbers increased modestly after. Since, the morphology is more similar to that of naturally differentiated adipocytes, we compared the expression level of adipocyte-specific genes (PPARγ, C/EBPα, LPL, C/EBPβ, C/EBPδ, and ADIPOQ) among CiADCs-3d, CiADCs-6d and natural adipocytes by qRT-PCR. In the results, the CiADCs showed similar adipocyte-specific gene expression patterns to adipocytes. These data indicate that BEFs have been transdifferentiated into adipocyte-like cells after 6 days of induction. Lipoprotein lipase (LPL), a key enzyme controlling lipid accumulation adipocyte, is highly expressed in fat cells (Bouraoui et al., 2012). PPARγ is abundant in adipose tissue and is induced before many adipocyte genes are transcribed, which plays an important role in adipocyte differentiation (Yang et al., 2017). C/EBPβ and C/EBPδ are regulatory factors in the early stage of adipocyte differentiation, which initiates adipocyte refinement by inducing the expression of PPARγ and C/EBPα (Darlington et al., 1998; Fajas et al., 1998). RVB increased protein expression of PPARγ and C/EBPα in the chemically-induced adipocytes (CiADCs-6d). These results suggested that PPARγ and C/EBPα play an important role in the mechanisms of conversion from BEFs to CiADCs.

In our sequencing data, the gene expression pattern of initial fibroblasts has changed significantly after experiencing the induction culture by small-molecule compound. Furthermore, analysis on gene enrichment proved that the genes of regulatory factors in the early stage of adipocyte differentiation (LPL, PPARγ, and C/EBPδ) (Macdougald and Lane, 1995) in CiADCs-3d were higher expression than that in CiADCs-6d. On the contrary, the later marker genes of adipocyte differentiation (C/EBPA, FAS, PCK2) (Macdougald and Lane, 1995) were more significantly upregulated in CiADCs-6d. This result not only indicated that CiADCs-6d tended to complete transdifferentiation of adipocytes, but also suggested that adipose-induced differentiation had started at the early stage of induction on the third day. According to GO analysis, there are many GO categories about adipocyte differentiation and lipid synthesis was enriched in biological processes and molecular function. It is important that highly expressed genes in CiADCs-3d/CiADCs-6d are enriched fat cell differentiation and cell fate commitment, which means that the cell fate of fibroblasts have changed. The upregulated expression trend of genes related to the adipocyte differentiation implied the trans-differentiation into adipocytes (Guo H et al., 2021). Gene set enrichment analysis (GSEA) showed the signaling pathway PPAR (Wenjing et al., 2016), PI3K-Akt (Wang et al., 2017), p53 (Molchadsky et al., 2013), and MAPK (Makdissy et al., 2015; Wu et al., 2017) of regulating adipocyte differentiation is already turned on by 3 days of induction. Also, these signaling pathways will still be detected when the cells were induced for 6 days. In addition, the fatty acid metabolism and fatty acid biosynthesis were clearly captured in CiADCs-3d. The upregulation FAS and LPL were key genes that regulate fatty acid metabolism (Kim and Spiegelman, 1996). Meanwhile, the fatty acid biosynthesis increases lipid accumulation (Horton and Shimomura, 1999).

In this study, the bovine adipocyte-like cells induced by pure small-molecule cocktails were appeared only for 6 days. In another research, the porcine embryonic fibroblasts were directly reprogrammed into adipocytes with the treatment of SB431542 and Thiazovivin. The adipocytes were observed for 10–15 days (Zhu et al., 2012). Afterwards, Sowa et al. (2021) revealed that human dermal fibroblasts can be directly converted into adipocytes by adding a single chemical compound, STK287794, for 14 days. Moreover, Xie’s team reported that RepSox in the fibroblast growth medium (MDI free) for 10 days was sufficient to induce adipogenesis in MEFs (Tu et al., 2019). However, we reported a method for transdifferentiating bovine fibroblasts into adipocytes and shortening the time of induction. This may be due to our culture system and combination of small-molecule compounds.

As a potent and selective TGFβRI/ALK5 inhibitor, Repsox inhibits the binding of ATP to ALK5 and the autophosphorylation of ALK5, and then inhibits TGF-β signaling (Tu et al., 2019; Guo Y et al., 2021). TGFβ signaling pathway regulates the differentiation program of a variety of cell types, including mesenchymal cell (Zhao and Hantash, 2011; Massagué and Xi, 2012; Grafe et al., 2017). R has crucial effect on the induction of mouse embryonic fibroblasts/sheep fibroblasts adipogenesis (Tu et al., 2019; Guo Y et al., 2021). It is worth noting that the TGF-β pathway has differing effects on pluripotency between species. Larsen et al. (2013) found when bovine embryonic fibroblasts were treated by a single small molecule (Repsox), only the gens of Actc1, BGLAP, and Fzd4 were significant upregulation without the generation of adipocytes. In our experiment, we showed for the first time that the bovine adipocyte-like cells which induced by the cocktail of small molecules (RVB) and N2B27 medium. We speculate that reprogramming of bovine fibroblasts to adipose differentiation was rapidly initiated by the synergistic effects of the small-molecule cocktail (RVB) compared to the single use of Repsox. Among the small-molecule combinations we selected, the VPA of HDAC inhibitors can greatly improve the reprogramming efficiency (Taniguchi et al., 2008; Yang et al., 2017). In addition, the research showed that VPA transforms cells into a state more suitable for altering cell fate through epigenetic modification (Huangfu et al., 2008). Unlike our study, a previous trial showed that TTNPB inhibits porcine pre-adipocyte differentiation by decreasing the expression of PPARγ and SREBP-1c (Brandebourg and Hu, 2005). However, the previous data in our study showed that the PPARG was upregulation. While TTNPB is a retinoic acid receptor (RAR) agonist that has been used for chemical reprogramming as a small-molecule booster (Chen et al., 2003). It was reported that TTNPB enhanced chemical reprogramming efficiency up to a factor of 40 times (Qin et al., 2017). TTNPB could induce the differentiation of embryonic carcinoma stem cells into adipocytes. Also, gene expression of the adipocyte marker of PPARγ and LPL were significantly upregulated (Bouchard and Paquin, 2013). The greater role of TTNPB is to help initiate reprogramming than to inhibit adipocyte differentiation in our experiments. On the other side, the N2B27 medium was reported that substantially boosts reprogramming (P Hou et al., 2013). This also helps explain the shortened time and increased efficiency of adipogenesis in our induction protocol. In this study, we demonstrated the feasibility of directly reprogramming bovine ear fibroblasts into adipocytes-like cells using RVB in N2B27 medium.

In conclusion, this study is the first to rapidly convert bovine ear fibroblasts into adipocyte-like cells *in vitro*. The induced adipocytes were very similar to natural fat cells. This result provides a cell model to further study the adipocyte differentiation and mechanism of adipocyte deposition of bovine and offers a new strategy to improve the beef quality.

## Data Availability

The datasets presented in this study can be found in online repositories. The names of the repository/repositories and accession number(s) can be found below: GEO Submission (GSE215910).
